# Critical Role of TLR7 Signaling in the Priming of Cross-Protective Cytotoxic T Lymphocyte Responses by a Whole Inactivated Influenza Virus Vaccine

**DOI:** 10.1371/journal.pone.0063163

**Published:** 2013-05-02

**Authors:** Natalija Budimir, Aalzen de Haan, Tjarko Meijerhof, Simke Waijer, Louis Boon, Emma Gostick, David A. Price, Jan Wilschut, Anke Huckriede

**Affiliations:** 1 Molecular Virology Section, Department of Medical Microbiology, University of Groningen, University Medical Center Groningen, Groningen, The Netherlands; 2 Bioceros BV, Utrecht, The Netherlands; 3 Institute of Infection and Immunity, Cardiff University School of Medicine, Cardiff, United Kingdom; University of Georgia, United States of America

## Abstract

Current influenza vaccines fail to induce protection against antigenically distinct virus strains. Accordingly, there is a need for the development of cross-protective vaccines. Previously, we and others have shown that vaccination with whole inactivated virus (WIV) induces cross-protective cellular immunity in mice. To probe the mechanistic basis for this finding, we investigated the role of TLR7, a receptor for single-stranded RNA, in induction of cross-protection. Vaccination of TLR7−/− mice with influenza WIV failed to protect against a lethal heterosubtypic challenge; in contrast, wild-type mice were fully protected. The lack of protection in TLR7−/− mice was associated with high viral load and a relative paucity of influenza-specific CD8+ cytotoxic T lymphocyte (CTL) responses. Dendritic cells (DCs) from TLR7−/− mice were unable to cross-present WIV-derived antigen to influenza-specific CTLs *in vitro*. Similarly, TLR7−/− DCs failed to mature and become activated in response to WIV, as determined by the assessment of surface marker expression and cytokine production. Plasmacytoid DCs (pDCs) derived from wild-type mice responded directly to WIV while purified conventional DCs (cDCs) did not respond to WIV in isolation, but were responsive in mixed pDC/cDC cultures. Depletion of pDCs prior to and during WIV immunization resulted in reduced numbers of influenza-specific CTLs and impaired protection from heterosubtypic challenge. Thus, TLR7 plays a critical role in the induction of cross-protective immunity upon vaccination with WIV. The initial target cells for WIV appear to be pDCs which by direct or indirect mechanisms promote activation of robust CTL responses against conserved influenza epitopes.

## Introduction

Influenza continues to represent a major global health burden [1]. Although vaccination is the cornerstone of control, current seasonal influenza vaccines offer only narrow protection. These vaccines induce neutralizing antibodies primarily against hemagglutinin (HA) and neuraminidase (NA), the highly variable surface glycoproteins of the virus [2], [3]. Thus, vaccine-induced immune responses are largely strain-specific and fail to protect against antigenically-drifted or newly-emerging shifted viruses with pandemic potential. These limitations underscore the need for novel, cross-protective influenza vaccines [4], [5].

Previously, we and others have shown that vaccination with whole inactivated virus (WIV) induces cross-protection against lethal heterosubtypic infection in mice, in contrast to subunit and split virion vaccines, which did not show any cross-protective capacity [6], [7]. This WIV-induced cross-protective effect is mediated principally by CD8+ cytotoxic T lymphocytes (CTLs). Indeed, the extent of protection has been shown to correlate with the number of influenza nucleoprotein (NP)-specific CTLs induced by vaccination. Furthermore, depletion of CD8+ cells results in loss of protection [6], [7]. Thus, WIV-induced cross-protection against heterosubtypic influenza infection in mice depends on the consequent CTL response, which operates to reduce viral load in the lungs after lethal dose challenge.

Naive CTLs are primed by dendritic cells (DCs), which present the respective peptide antigen in a major histocompatibility complex (MHC) class I-restricted manner and provide additional co-stimulation [8], [9]. Antigens produced in the cytosol of DCs, such as viral proteins in the case of infection, have direct access to the MHC class I presentation pathway. Other antigens, such as those present in WIV, can access this pathway through a process known as cross-presentation [10], [11]. Only mature and activated DCs are licensed for successful cross-presentation of exogenous antigens [11]. Accordingly, proper activation of DCs is considered to be a key event in the induction of specific CTL responses to non-replicative antigens like WIV [12], [13]. Previously, we showed that WIV activates bone marrow-derived conventional (cDCs) and plasmacytoid DCs (pDCs) and that activation of the latter is dependent on engagement of Toll-like receptor 7 (TLR7) by the single-stranded (ss) viral RNA in WIV [14], [15]. In the present study, we investigated the role of TLR7 signaling in the induction by WIV of CTL responses and cross-protective immunity against lethal heterosubtypic challenge.

Strikingly, TLR7-deficient (TLR7−/−) mice were not protected from lethal H1N1 challenge by vaccination with H5N1 WIV, whereas wild-type (*wt*) mice were fully protected. The absence of cross-protection in vaccinated TLR7−/− mice correlated with a lack of NP-specific CTLs. *In vitro*, bone marrow-derived TLR7−/− DCs were incapable of presenting WIV-derived antigens to primed NP-specific CTLs and failed to undergo maturation and activation in response to WIV exposure. Sorted bone marrow-derived plasmacytoid DCs (pDCs), but not sorted conventional DCs (cDCs), from *wt* mice responded directly to WIV stimulation by surface marker (MHC class I, CD86, CD80, CD40) upregulation and cytokine (IFNα and IL12) secretion. In mice depleted of pDCs during immunization, CTL induction and protection against heterosubtypic challenge were impaired. Thus, TLR7 triggering is essential for the successful induction of cross-protective cellular immunity by WIV. The primary target cells for the vaccine are pDCs, which appear to play an important role in the induction of virus-specific CTLs.

## Materials and Methods

### Ethics Statement

All mouse experiments were performed in strict accordance with Dutch legislation on animal experiments (“Wet op de dierproeven”, 1977; modified in 1996 with implementation of the European guidelines 86/609/EEG and “Dierproevenbesluit 1985”) and approved by the Ethics Committee on Animal Research of the University Medical Center Groningen (Permit number: 5101B).

### Virus Strains and Vaccines

Egg-derived A/PR/8/34 (H1N1) virus and egg-derived A/New Caledonia/IVR 116 (H1N1) virus were kind gifts from Solvay Biologicals (Weesp, The Netherlands); these viruses were further amplified on eggs according to standard procedures. A/NIBRG-14, a genetic reassortant of A/PR/8/34 and A/Vietnam/1194/2004 (H5N1), was obtained from NIBSC (Potters Bar, UK) and cultured on Madine-Darby Canine Kidney (MDCK) cells. WIV vaccine was prepared by inactivation of NIBRG-14 virus for 24 hr with 0.1% β–propiolactone (BPL; Acros Organics, Geel, Belgium) at room temperature, followed by dialysis for 24 hr against HNE buffer (5 mM HEPES, 150 mM NaCl, 0.1 mM EDTA, pH 7.4).

Inactivation of the virus was tested by performing serial passages on eggs, according to the protocol published in the European Pharmacopeia [16]. Specifically, one vaccine dose containing 20 µg of total viral protein was injected into the allantoic cavity of each of 20 fertilized eggs and eggs were incubated for 3 days at 33°C. Subsequently, 1 ml aliquots of allantoic fluid from each egg were pooled, and 200 µl was inoculated into each new egg. This passage was repeated once more. After the last passage, allantoic fluid was harvested and the absence of replicative virus was demonstrated by a hemagglutination test, as described elsewhere [17].

### Mice

Female C57Bl/6 and TLR7−/− mice, 8–10 weeks old, were used in immunization and challenge studies. C57Bl/6 mice were vendor sourced (Harlan, The Netherlands) and TLR7−/− mice (a kind gift from S. Akira and C. Reis e Sousa) were bred at the animal facility of the University Medical Center Groningen (Groningen, The Netherlands). All mice were kept under SPF conditions in conventional cages and had access to food and water *ad libitum*.

All experimental handeling of mice, such as vaccination, blood sampling, challenge and euthanasia, were performed under isoflurane anesthesia. Animals were euthanized at the indicated time or when weight loss exceeded 20%. Euthanasia was performed by heart puncture under isoflurane anesthesia.

### Vaccination and Heterosubtypic Challenge

C57Bl/6 and TRL7−/− mice (5–6 per experimental group) were vaccinated twice (days 0 and 21) with 20 µg of NIBRG-14 (H5N1) WIV administered subcutaneously (s.c.) or similarly mock-vaccinated with HNE buffer as described previously [6]. One week after the booster immunization, mice were anesthetized and inoculated intranasally with 100 PFU of PR8 (H1N1) in 40 µl saline. After the challenge, mice were monitored daily for body weight change. Body weight loss of more than 20% was considered an indication for euthanasia. On day 6 post-challenge, 6 mice from each group were euthanized, and lungs and BAL were collected for analysis of virus titers, tetramer staining and granzyme B measurements. Mice that did not lose more than 20% of their body weight were euthanized on day 14 post-challenge for analysis of virus titers and immunological parameters.

Protocols for virus titration in the lung tissue and tetramer staining of lung-derived lymphocytes are described elsewhere [6]. Granzyme B content in BAL was measured by ELISA (R&D Systems, Abingdon, UK) according to the manufacturer’s protocol.

### *In vivo* Cytotoxicity Assay

C57Bl/6 and TLR7−/− mice were vaccinated twice (days 0 and 21) with either 25 µg of NIBRG-14 WIV (s.c.) or 400 HAU of A/New Caledonia live virus (i.p.); HNE mock-vaccinated (s.c.) mice served as negative controls. On days 7 and 8 after the booster immunization, an *in vivo* cytotoxicity assay was performed as described previously [6].

### *In vitro* Reactivation of Influenza-specific CTLs and Tetramer Staining

Naive C57Bl/6 mice were primed by intraperitoneal injection of 400 HAU of A/New Caledonia live virus. Three weeks later, spleens were dissected and collected on ice in complete Iscove’s modified Dulbecco’s medium (IMDM; Invitrogen, Breda, The Netherlands). Splenocytes were isolated by homogenizing spleens through cell strainers (BD Biosciences) and resuspended in medium. After 10 min centrifugation (350×g) at 4°C, erythrocytes were removed by lysis using ACK buffer (0.15 mM NH_4_Cl, 10 mM KHCO_3_, 0.1 mM EDTA, pH 7.4). Subsequently, 10^7^ splenocytes were cocultured with 10^6^ *wt* or TLR7−/− DCs previously pulsed with 5 µg/ml of WIV. As a control, 10^6^ TLR7−/− DCs were pulsed with 5 µg/ml WIV supplemented with 10 µg/ml ODN1826 CpG (InvivoGen, Toulouse, France). Cocultures were performed in T25 flasks in complete IMDM supplemented with 10 U/ml IL2 (PeproTech, London, UK). After 7 days, cells were centrifuged, resuspended in PBS supplemented with 1% bovine serum albumin (BSA) and 5 mM EDTA, and stained for 45 min at 4°C with anti-mouse CD8**α** α-allophycocyanin (ImmunoSource, Halle-Zoersel, Belgium) and influenza NP_366–374_ tetramer-phycoerythrin (PE). Tetramers were produced by conjugation of soluble biotinylated NP_366–374_/H-2Db monomeric protein at a 4∶1 molar ratio with PE-conjugated streptavidin as described elsewhere [18]. Dead cells were excluded from flow cytometric analyses using 7AAD (ImmunoSource).

### Culture, Maturation and Activation of DCs

DCs were generated from murine bone marrow isolated from tibia and femur of C57Bl/6 (*wt* DCs) and TLR7−/− (TLR7−/− DCs) mice, and cultured for 8 days in IMDM supplemented with 10% heat-inactivated fetal bovine serum (FBS), 1% antibiotics, 0.1% β-mercaptoethanol (Invitrogen) and 200 ng/ml Flt3L (R&D Systems).

Upregulation of surface marker expression was measured by flow cytometry after pulsing DCs with either WIV (5 µg/ml), CpG (10 µg/ml) or culture medium. A total of 3×10^6^ DCs were incubated at 1×10^6^ cells/ml with each stimulating agent at 37°C in complete IMDM. One portion of 3×10^6^ *wt* DCs was incubated for 3 hr with the TLR7 antagonist IRS661 (5 µg/ml; Eurogentec, Maastricht, The Netherlands), then pulsed with WIV (5 µg/ml). Cells were stained with directly conjugated anti-mouse monoclonal antibodies (ImmunoSource) specific for MHC class I, CD86, CD80 and CD40 according to a standard protocol. Flow cytometric analysis was conducted using a FACSCalibur (BD Biosciences).

Production of IL12 was measured in cell culture supernatants by ELISA (ImmunoSource) according to the manufacturer’s protocol. Detection of IFNα was performed according to an ELISA protocol published elsewhere [14].

### pDC/cDC Sorting

After 8 days in culture, DCs were stained with anti-mouse CD11b-PECy7, anti-mouse B220-eFluor450 and anti-mouse CD11c-PE (ImmunoSource) and sorted using a FACSAria (BD Biosciences) cell sorter to separate pDC and cDC populations. pDCs were identified as B220+CD11c+CD11b− cells and cDCs were identified as B220-CD11c+CD11b+ cells [19]. After sorting, cells were collected in FBS-coated FACS tubes at a final concentration of 70% FBS.

### pDC Depletion

pDC depletion was performed by administration of purified pDC-depleting monoclonal antibody (clone 120G8) [20]. On 3 consecutive days (1 day pre-vaccination, day of vaccination and 1 day post-vaccination) mice were injected i.p. with 300 µg of the depletion antibody. This pDC depletion procedure was repeated once more during the booster vaccination (days 20–22). Control mice received the same amount of isotype control antibody or PBS. The efficacy of pDC depletion was determined in bone-marrow derived cells 1 day after the last depletion dose was administered. pDCs were identified using two different staining strategies (CD11b−CD11c+PDCA-1+ and CD11b−CD11c+B220+) followed by flow cytometric analysis. Results of depletion are depicted in the figure S1.

### Statistical Methods

Statistical differences between different treatment groups were analyzed using the Mann-Whitney U-test with a confidence interval of 95%. A value of p<0.05 was considered statistically significant (depicted as an asterisk).

## Results

### Role of TLR7 Signaling in WIV-induced Heterosubtypic Cross-protection

**Figure 1:**
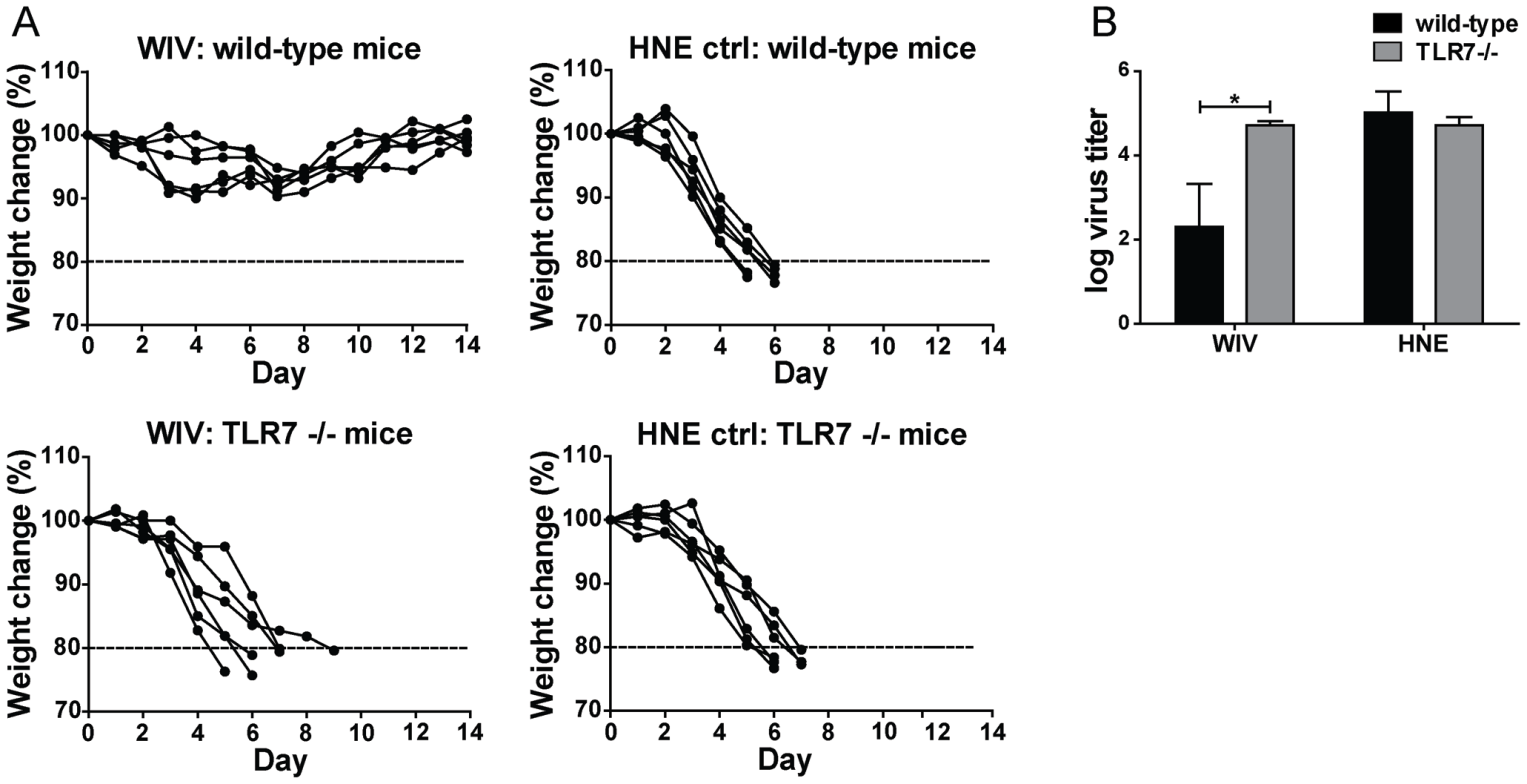
Heterosubtypic cross-protection induced by WIV depends on TLR7 activation. *Wt* and TLR7−/− mice were either vaccinated twice (on days 0 and 21) subcutaneously (s.c.) with 20 µg of H5N1 WIV or similarly mock-vaccinated with HNE buffer. A lethal heterosubtypic challenge with H1N1 virus was administered one week later. (A) After challenge, mice were monitored daily over a period of 14 days for body weight change. Body weight loss of more than 20% was an indication for euthanasia (dashed line). (B) Virus titers in the lungs of *wt* (black bars) and TLR7−/− (white bars) mice were measured 6 days post-challenge. Bars represent mean±SEM of 3 mice per group. *p<0.05; Mann-Whitney U-test.

To investigate the role of TLR7 signaling in WIV-induced heterosubtypic protection, we vaccinated *wt* and TLR7−/− mice with two doses of WIV derived from A/H5N1 virus (NIBRG-14). One week after the second immunization, mice were exposed to a lethal challenge with A/H1N1 virus (PR8) and monitored for 14 days or euthanized when their body weight loss exceeded 20%. All of the vaccinated *wt* mice survived the lethal heterosubtypic challenge without significant weight loss. In contrast, all of the vaccinated TLR7−/− mice, together with the mock-vaccinated controls, had lost more than 20% of their body weight by day 9 post-challenge and were euthanized (Fig. 1A).

On day 6 post-challenge, we assessed virus clearance from the lungs of the challenged mice. As expected, lung virus titers were reduced by almost 3 logs in WIV-vaccinated *wt* mice compared to mock-vaccinated mice. In contrast, viral titers in the lungs of TLR7−/− mice vaccinated with WIV were high and similar to those measured in the lungs of mock-vaccinated controls (Fig. 1B).

Thus, TLR7 is critically involved in WIV-induced control of virus growth in the lungs and protection from lethal heterologous virus challenge.

### Role of TLR7 Signaling in WIV-mediated Induction of Influenza-specific CTLs

**Figure 2:**
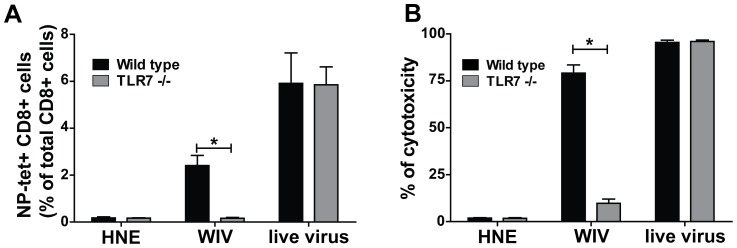
Influenza-specific cytotoxicity *in vivo* depends on TLR7 activation. *Wt* (black bars) and TLR7−/− (white bars) mice received two doses (on day 0 and 21) of WIV (25 µg; s.c.), replicative virus (400 HAU; i.p.) or HNE buffer (s.c.). One week later, mice were injected through the orbital vein with a 1∶1 mixture of NP- and OVA-pulsed target cells, differentially labeled with high and low concentrations of CFSE, respectively. NP_366–374_ tetramer-positive CD8+ T cells (A) and cytotoxicity expressed as % of killed target cells (B) were measured by tetramer staining and flow cytometric analysis of splenocytes isolated from immunized mice 13 hours after target cell injection. Gating was based on the forward-side scatter pattern and dead cells (7AAD+) were excluded. Finally, gates were set on CD8+tetramer+ cells. Results are representative of 3 independent experiments. Bars represent mean±SEM of 5 mice per group. *p<0.05; Mann-Whitney U-test.

Previously, we showed that WIV-induced cross-protection of mice is mediated primarily by influenza-specific CTLs. Therefore, we now investigated whether the observed lack of cross-protection in TLR7−/− mice was due to an impaired CTL response in these animals. *Wt* and TLR7−/− mice were immunized twice with H5N1 WIV. Seven days after the second immunization, CTLs specific for the NP_366–374_ epitope were quantified by peptide-MHC class I tetramer staining. As depicted in Figure 2A, tetramer-positive CD8+ cells were detected in the spleens of WIV-immunized *wt* mice but not in the spleens of WIV-immunized TLR7−/− mice. The mutant mice were not impaired in terms of their ability to generate CTLs *per se*, as intraperitoneal injection of live influenza virus resulted in similarly high numbers of NP_366–374_ tetramer-positive cells in both *wt* and TLR7−/− mice (Fig. 2A). Live influenza virus has the capacity to activate innate immune receptors such as RLRs (e.g. RIG-I), NLRs (e.g. NLRP3) and TLR3, and thus does not necessarily require TLR7 to enable CTL activation [21]–[24].

In addition, we assessed the induction of influenza-specific CTL activity by measuring the level of *in vivo* cytotoxicity after immunization with WIV. To this end, we injected CFSE-labeled, NP_366–374_ peptide-loaded target splenocytes from *wt* donor mice into recipient *wt* and TLR7−/− mice immunized with H5N1 WIV. One day later, target cell survival was scored in the spleen. Figure 2B depicts the cytotoxic activity of influenza-specific CTLs against peptide-loaded target cells. Immunization of *wt* mice with WIV induced high levels of cytotoxicity in these animals. In contrast, lysis of target cells was not detected in TLR7−/− mice after immunization with WIV. When CTLs were primed by infection with live virus, both *wt* and TLR7−/− mice displayed similar levels of cytotoxicity against peptide-loaded target cells.

**Figure 3:**
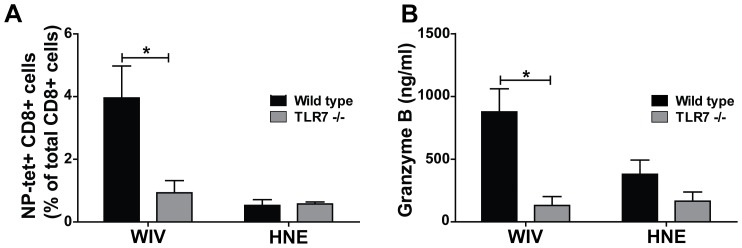
Local activation of influenza-specific CTLs in the lungs depends on TLR7 activation. (A) NP_366–374_ tetramer-positive CD8+ T cells in the lungs of *wt* (black bars) and TLR7−/− (white bars) mice vaccinated with WIV were quantified on day 6 post-challenge. Gating strategy was as described in the legend to Fig. 2 (B) Local production of granzyme B in the lungs of *wt* (black bars) and TLR7−/− (white bars) mice vaccinated with WIV was measured on day 6 post-challenge by ELISA performed on BAL samples. Bars represent mean±SEM of 3 mice per group. *p<0.05; Mann-Whitney U-test.

Next, we assessed the induction of CTL activity in the lungs of mice after vaccination and heterosubtypic challenge. To this end, *wt* and TLR7−/− mice were vaccinated twice with H5N1 WIV and, one week after the booster dose, exposed to a lethal heterosubtypic challenge with H1N1 virus. Six days later, the mice were sacrificed and local cytotoxic immune responses were evaluated in lung tissue and bronchoalveolar lavage (BAL) by NP_366–374_ tetramer staining and granzyme B measurement, respectively. Substantial numbers of tetramer-positive CD8+ cells were detected in the lungs of *wt* mice vaccinated with WIV (Fig. 3A). In contrast, very few tetramer-positive CD8+ cells were detected in the lungs of WIV-vaccinated TLR7−/− mice, numbers barely exceeding those observed in the lungs of mock-vaccinated control mice. In parallel, granzyme B was absent in BAL collected from the vaccinated TLR7−/− mice, while high levels were detected in BAL from their vaccinated *wt* counterparts (Fig. 3B).

Collectively, these data indicate that TLR7 activation is crucial for the induction of influenza-specific CTL activity upon vaccination with WIV.

### Role of TLR7 Signaling in Cross-presentation of WIV-derived Antigen

**Figure 4:**
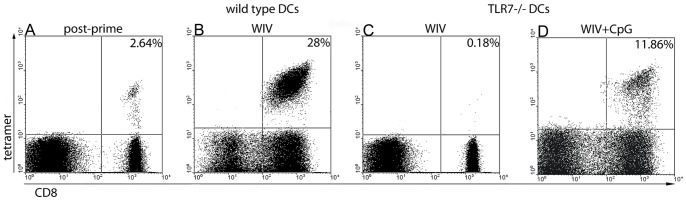
*In vitro* restimulation of primed influenza-specific CD8+ T cells by DCs depends on TLR7 activation. *Wt* mice were primed with 400 HAU of live influenza A/New Caledonia virus (i.p.). NP_366–374_ tetramer-positive CD8+ T cells were quantified in the spleen 3 weeks later (A). Splenocytes from primed mice were cocultured for 7 days with either murine bone marrow-derived *wt* DCs (B), TLR7−/− DCs pulsed with WIV (C), or TLR7−/− DCs pulsed with WIV+CpG (D). Gates were set on viable cells based on the forward/side scatter profile and exclusion of 7AAD+ cells. Frequencies of NP_366–374_ tetramer-positive CD8+ T cells are shown as a percentage of the total CD8+ T cell population. Results are representative of 4 independent experiments.

Originating from a non-replicative vaccine, WIV-derived antigens must be presented to CD8+ T cells by professional antigen-presenting cells (APCs), specifically DCs, by a process of cross-presentation. To determine the role of TLR7 in cross-presentation of WIV-derived antigens (e.g. epitopes derived from NP), we investigated the capacity of WIV-pulsed bone marrow-derived TLR7−/− DCs to induce *in vitro* restimulation of influenza-specific CD8+ T cells. The degree of restimulation was assessed by enumeration of NP_366–374_ tetramer-positive cells by flow cytometry before and after *in vitro* coculture. Stimulation of primed CD8+ T cells for 7 days with *wt* DCs exposed to WIV induced massive expansion of the NP_366–374_ tetramer-positive CD8+ T cell population (Fig. 4A & 4B). In contrast, coculture of primed CD8+ T cells with TLR7−/− DCs pulsed with WIV did not induce proliferation of NP_366–374_ tetramer-positive CD8+ T cells (Fig. 4C). To demonstrate that the absence of *in vitro* CTL restimulation by WIV-pulsed TLR7−/− DCs was not an intrinsic defect of TLR7−/− cells, we incubated these cells simultaneously with WIV and a TLR9 ligand (CpG oligodeoxynucleotides). As shown in Figure 4D, CpG-mediated TLR9 activation could compensate for the absence of TLR7 signaling, resulting in efficient expansion of NP_366–374_ tetramer-positive CD8+ T cells. These data demonstrate a crucial role for TLR7 in the cross-presentation of WIV-derived antigens, specifically NP, by DCs.

### Role of TLR7 Signaling in WIV-induced Maturation and Activation of DCs

It is established that only DCs with a mature and activated phenotype are capable of effective antigen presentation [11], [25]. To investigate the role of TLR7 signaling in the induction of such a phenotype, we compared the responses of *wt* and TLR7−/− bone marrow-derived DCs to stimulation with WIV. In addition, we investigated WIV-induced maturation and activation of *wt* DCs in which TLR7 signaling was blocked using a specific antagonist (IRS661).

**Figure 5:**
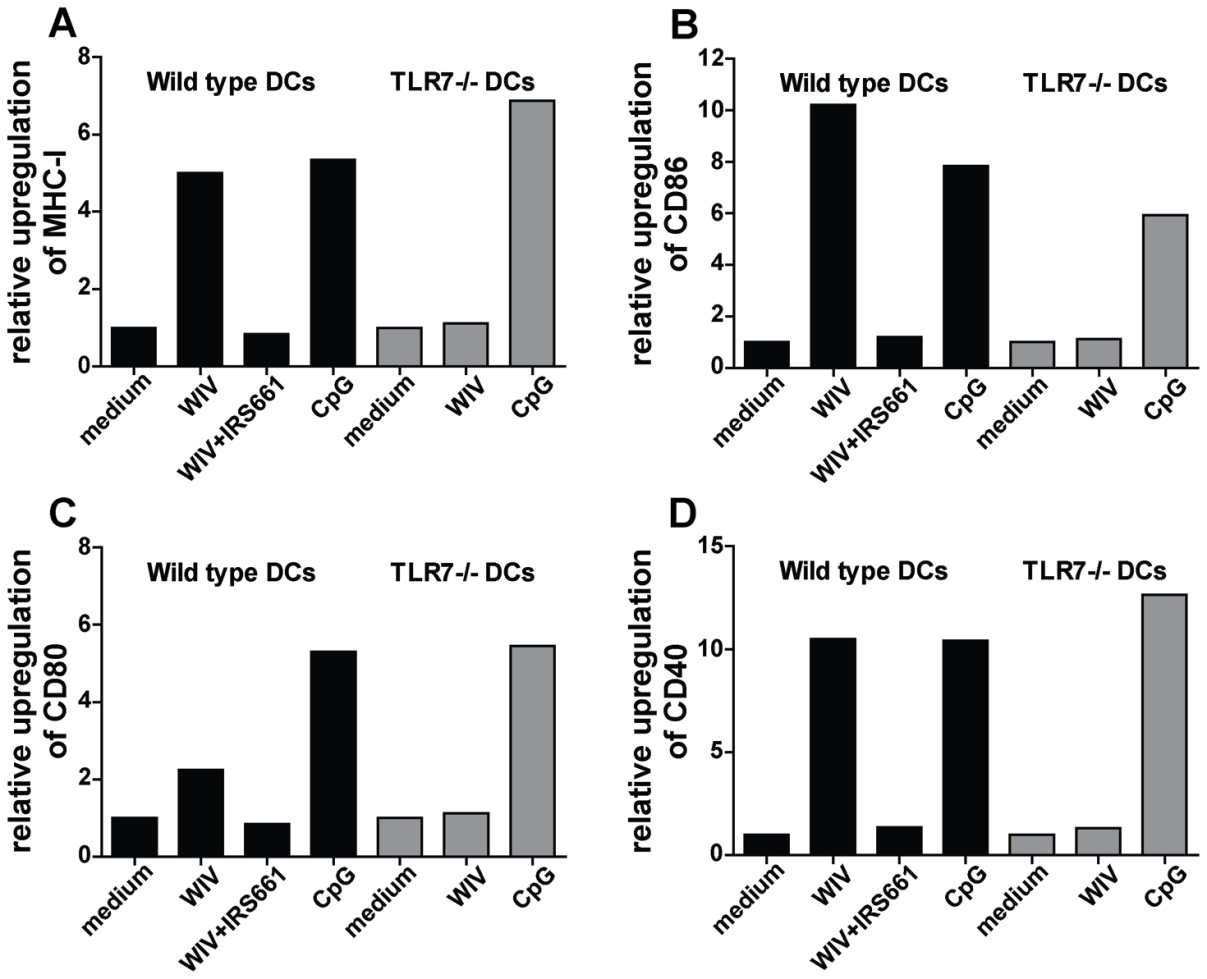
WIV-induced maturation of DCs depends on TLR7 activation. Murine bone marrow-derived *wt* (black bars) and TLR7−/− (gray bars) DCs were pulsed for 24 hr with WIV, WIV+IRS661, CpG or culture medium. Expression of the following maturation markers was measured by flow cytometry: MHC class I (A), CD86 (B), CD80 (C) and CD40 (D). Gates were set on viable cells based on the forward/sideward scatter profile and exclusion of 7AAD+ cells. Fold changes of mean fluorescence intensity are depicted. Results are representative of 3 independent experiments in each of which 100000 cells were analyzed.

As depicted in Figure 5, *wt* DCs exhibited a marked upregulation of surface maturation markers (MHC class I, CD86, CD80 and CD40) in response to stimulation with WIV. In contrast, expression of these markers remained unchanged in parallel experiments with TLR7−/− DCs. Consistent with this observation, inhibition of TLR7 completely abolished WIV-induced surface marker upregulation in *wt* DCs. The absence of a response in TLR7−/− DCs was not due to an intrinsic defect of these cells, as activation with the TLR9 ligand CpG induced efficient maturation of TLR7−/− DCs.

**Figure 6:**
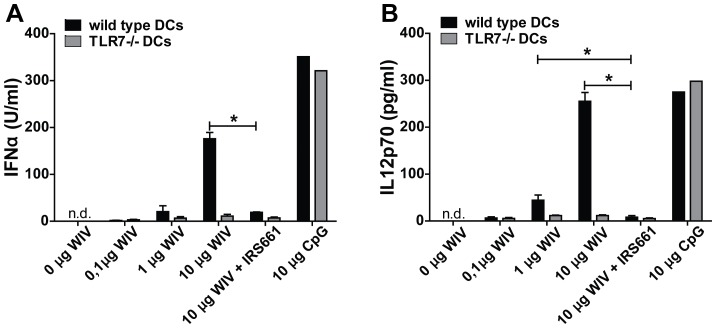
WIV-induced production of IFN-α and IL-12 depends on TLR7 activation. Murine bone marrow-derived *wt* (black bars) and TLR7−/− (gray bars) DCs were pulsed for 24 hr with different amounts of WIV (0.1, 1 and 10 µg), WIV+IRS661, CpG or culture medium. Secreted concentrations of IFNα (A) and IL12 (B) were measured by ELISA. All measurements were performed in triplicate; bars represent mean±SEM. Results are representative of 3 independent experiments. *p<0.05; Mann-Whitney U-test.

In addition, we measured IFNα and IL12 secretion to determine the activation status of DCs (Fig. 6). Both cytokines were secreted in a dose-dependent manner by *wt* DCs, but not TLR7−/− DCs, after stimulation with WIV. Consistently, blockade of TLR7 signaling in *wt* DCs completely inhibited the induction of IFNα and IL12. TLR7−/− DCs were capable of producing IFNα and IL12 when stimulated with CpG demonstrating the general capacity to produce cytokines.

These results demonstrate that TLR7 signaling controls the maturation and activation of DCs upon exposure to WIV.

### Role of TLR7 in Activation and Maturation of Plasmacytoid DCs (pDCs) and Conventional DCs (cDCs)

**Figure 7:**
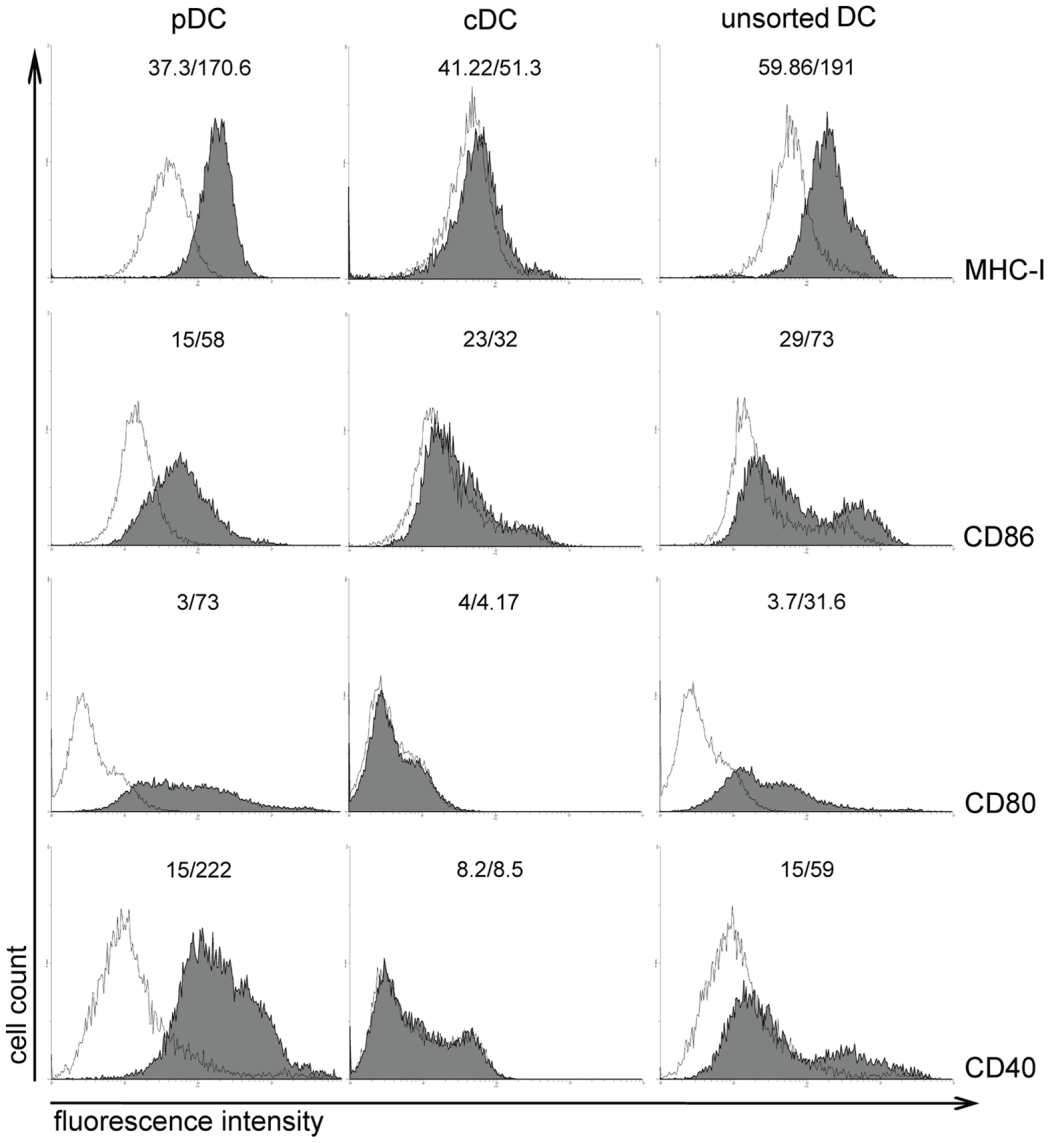
WIV induces maturation of pDCs but not cDCs. Bone marrow-derived pDCs (B220+CD11c+CD11b−), cDCs (B220-CD11c+CD11b+) and unsorted DCs were pulsed for 24 hr with WIV (gray histogram) or culture medium (white histogram). Upregulation of surface maturation marker expression was measured by flow cytometry. Gates were set on viable cells based on the forward/side scatter profile and exclusion of 7AAD+ cells. Numbers above each histogram represent the mean fluorescence intensity measured in medium-stimulated and WIV-stimulated DCs, respectively. Results are representative of 3 independent experiments.

**Figure 8:**
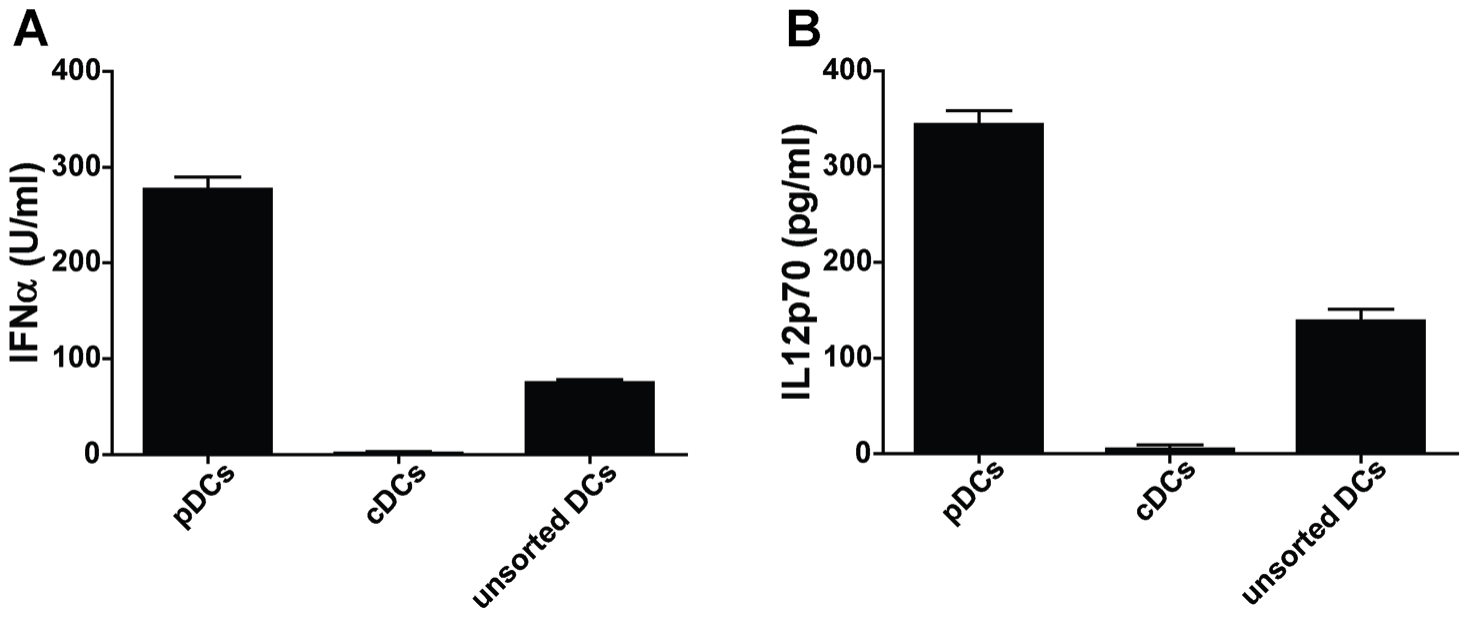
WIV induces cytokine production in pDCs but not cDCs. Bone marrow-derived pDCs (B220+CD11c+CD11b−), cDCs (B220-CD11c+CD11b+) and unsorted DCs were pulsed for 24 hr with WIV or culture medium. Secreted concentrations of IFNα (A) and IL12 (B) were measured by ELISA. Bars represent mean±SEM of triplicates corrected for the medium control. Results are representative of 3 independent experiments.

Next, we assessed the capacity of two major DC subpopulations, pDCs and cDCs, to respond to WIV stimulation. Based on the expression of characteristic surface markers, pDCs (B220+CD11c+CD11b−) and cDCs (B220-CD11c+CD11b+) from bone marrow cultures of both *wt* and TLR7−/− mice were sorted by flow cytometry; maturation and activation of these separate cell populations was then assessed in response to WIV stimulation. In sorted cultures, *wt* pDCs responded to WIV stimulation by upregulation of surface maturation markers (Fig. 7, left panels). In contrast, sorted cDCs were largely unresponsive to WIV (Fig. 7, middle panels). Similarly, *wt* pDCs were activated by WIV to secrete IFNα and IL12; no cytokine secretion was observed in parallel experiments with *wt* cDCs (Fig. 8). In unsorted cultures, a degree of maturation and activation was observed in both pDCs and cDCs as indicated by a general shift of the cell populations to higher fluorescence intensities, most pronounced for MHC-I and CD80 (Fig. 7, right panels). As expected, surface maturation marker upregulation and cytokine secretion were not observed in either sorted or unsorted cultures of TLR7−/− DCs (data not shown).

These results demonstrate that pDCs, but not cDCs, are activated directly in response to WIV stimulation and acquire a mature phenotype, in a TLR7-dependent manner. In contrast, cDC maturation is induced only indirectly, most probably in response to cytokines (e.g. IFNα) released by activated pDCs.

### Role of pDCs in CTL Induction upon Immunization with WIV *in vivo*

In order to obtain further insight into the role of pDCs in CTL induction by WIV *in vivo,* we depleted mice from pDCs by administering the pDC-specific antibody 120G8 prior to and during immunization of mice. This treatment reduced the number of pDCs (defined as PDCA-1+CD11c+CD11b− or as B220+CD11c+CD11b−) in bone marrow by a factor of 2–2.5 (Fig. S1). The level of depletion in spleen, assumed to be similar to or even higher than in bone marrow, could not be determined due to the notoriously low numbers of pDCs in spleen and blood of C57Bl/6 mice [26].

**Figure 9:**
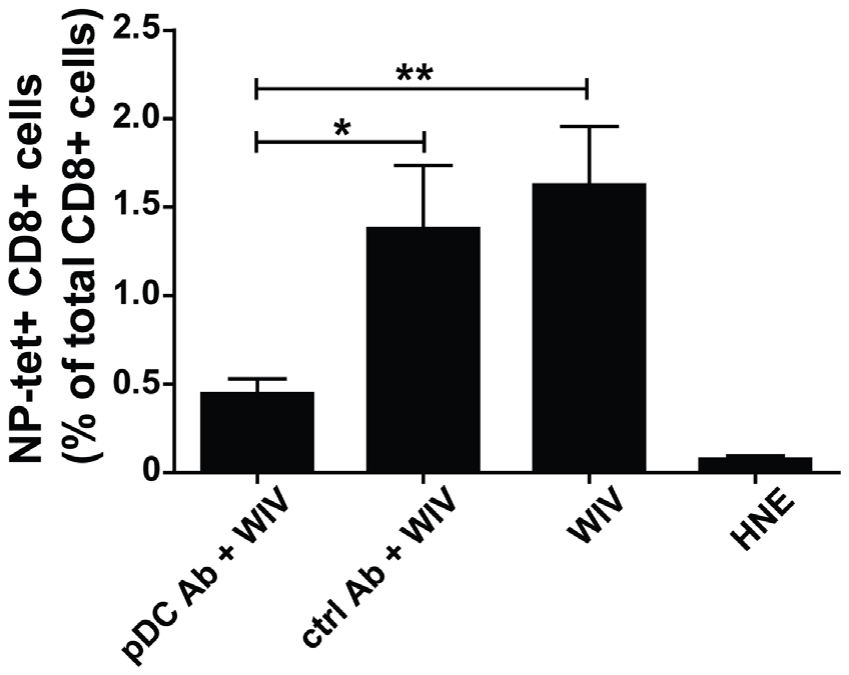
pDCs play a role in *in vivo* induction of CTLs by WIV. Mice were injected on day 0,1, and 2 with the pDC-depleting antibody 120G8 (pDC Ab), a non-specific isotype control (ctrl Ab) or left untreated. On day 1 they were immunized s.c. with 20 µg of H5N1 WIV or mock-vaccinated with HNE buffer. The procedure of pDC depletion and immunization was repeated on days 20–22. One week later the level of NP-specific CD8+ T cells in peripheral blood was evaluated by tetramer staining. Gating was based on the forward-side scatter pattern and dead cells (7AAD+) were excluded. Finally, gates were put on CD8+tetramer+ cells. Bars represent mean±SEM of 5 mice per group. *p<0.05, **p<0.01; Mann-Whitney U-test.

**Figure 10:**
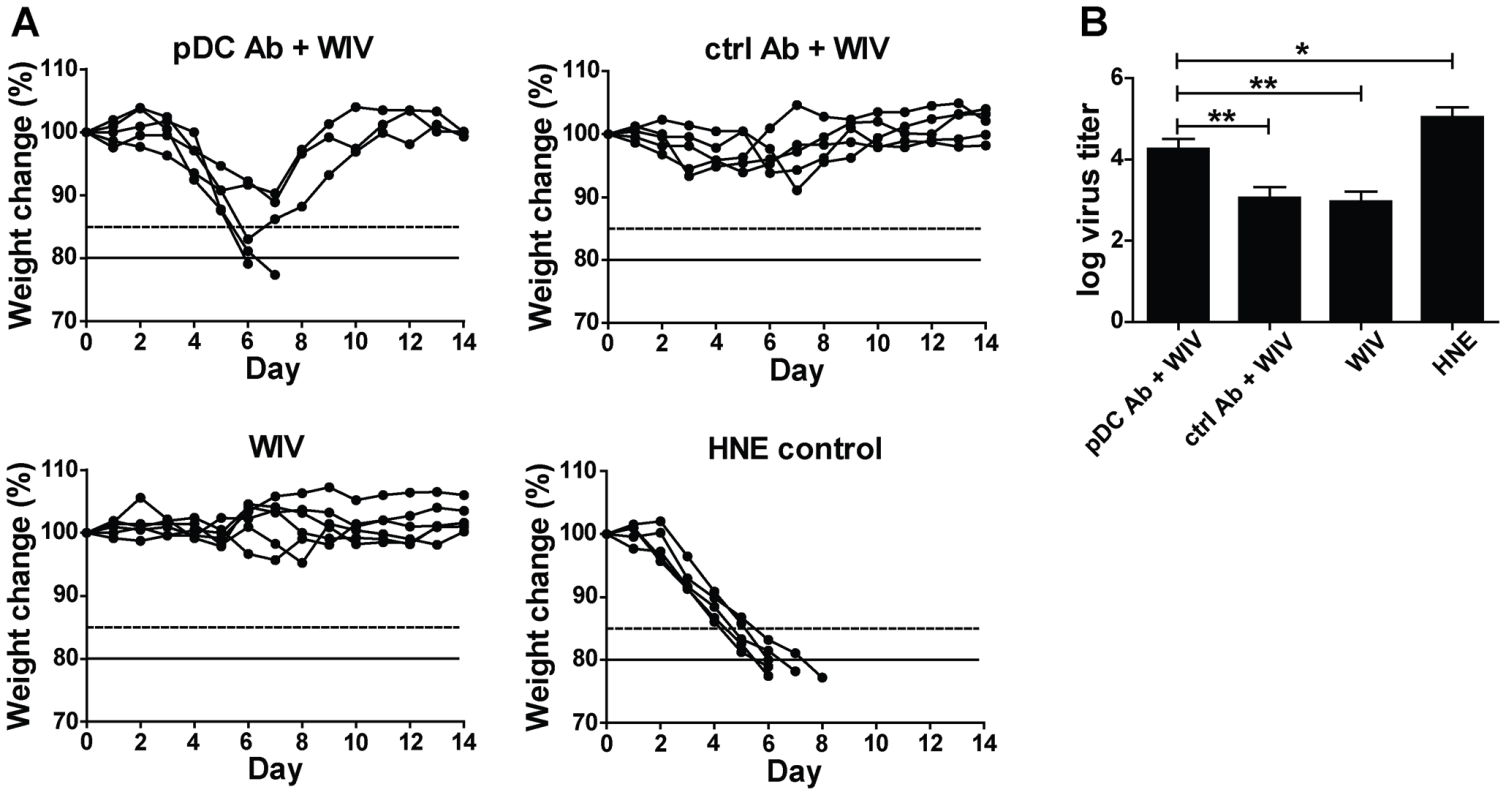
pDCs are important for WIV-afforded protection against heterologous challenge. Mice were depleted of pDCs and immunized as described in the legend to Fig. 9. One week later they received a lethal heterosubtypic challenge with H1N1 virus. (A) Weight was monitored daily for a period of 14 days. Weight loss of 15% (dashed line) was considered severe and weight loss of 20% (solid line) was an indication for euthanasia. Individual mice are depicted. (B) 5 mice per treatment group were sacrificed on day 6 after challenge and lung virus titers were determined. Bars represent mean±SEM. *p<0.05, **p<0.01; Mann-Whitney U-test.

Mice which received pDC-depleting antibody developed significantly lower numbers of NP-specific CD8+ T cells in response to immunization with H5N1 WIV than non-treated mice or mice which received an isotype control antibody instead of 120G8 (Fig. 9). When challenged with a lethal dose of H1N1 virus, pDC-depleted mice showed significant weight loss despite the immunization and two of the five mice had to be euthanized since they lost more than 20% of body weight (Fig. 10A). In contrast, non-treated mice or mice treated with a control antibody prior to and during WIV immunization did not lose weight. As expected, loss of weight was severe and rapid in non-immunized mice and reached 20% in all animals by day 6–8. The observed weight loss correlated with lung virus titers: pDC-depleted immunized mice demonstrated a significantly higher viral load in the lungs than non-depleted immunized mice (Fig. 10B). Yet, as compared to non-immunized control mice virus titers in immunized pDC-depleted mice were significantly reduced though to a moderateextent.

Taken together these results indicate that pDCs play a crucial role in the induction of CTLs by immunization with WIV *in vivo*.

## Discussion

Here, we describe the role of the innate pattern recognition receptor TLR7 in WIV-mediated induction of CTL responses and heterosubtypic cross-protection against influenza infection. In contrast to *wt* mice, TLR7−/− mice could not be protected from heterosubtypic virus challenge by immunization with WIV, indicating that TLR7 signaling plays a critical role in WIV-induced cross-protective immunity. The lack of protection in TLR7−/− mice was associated with the absence of specific WIV-induced CTL responses, which was linked to impaired cross-presentation of WIV-derived antigens by TLR7−/− DCs. Consistently, TLR7−/− DCs were unresponsive to WIV. In contrast, WIV induced the maturation and activation of *wt* DCs; pDCs responded directly while cDCs responded in an indirect way. pDCs also played a critical role in the induction of WIV-induced CTL responses *in vivo.* These results suggest that activation of TLR7 by WIV, particularly in pDCs, is crucial for effective priming of influenza-specific CTLs and, consequently, for the heterosubtypic cross-protection observed with this vaccine.

To our knowledge, the present study is the first to show that TLR7 signaling plays an essential role in CTL priming by immunization with WIV, and thus in the generation of heterosubtypic immunity. Other authors have addressed the role of TLR7 signaling in influenza WIV-induced homosubtypic protection. For example, Koyama *et al*. observed decreased survival in TLR7−/− mice compared to *wt* mice after immunization with WIV derived from A/New Caledonia/20/99 (an H1N1 variant circulating in the last decade) and challenge with A/PR/8/34 (an early H1N1 virus) [27], [28]. This lack of protection against a homosubtypic virus in WIV-immunized TLR7−/− mice was explained by hampered antibody responses. Other immune mechanisms were not investigated in these studies. Extending the study of Koyama *et al*., we demonstrate here that the lack of protection against heterosubtypic infection in WIV-immunized TLR7−/− mice is closely correlated with hampered cellular immune responses, in particular an almost complete absence of influenza-specific CTLs.

Our study shows that, in the absence of TLR7 signaling, CTL induction by WIV is largely abolished. In contrast, live influenza virus was capable of inducing a strong antigen-specific CTL response even in TLR7−/− mice, consistent with previous observations (27). It is known that live virus can activate innate receptors other than TLR7, such as RIG-I, which could result in DC activation and, consequently, the induction of specific CTL responses [27], [28]. Indeed, recent evidence suggests that RIG-I activation requires live virus replication in the cell [23], [29]. This finding explains the absence of RIG-I activation by non-replicative WIV and, by extension, the strict TLR7-dependence of WIV-induced CTL responses.

The natural ligand of TLR7 is single stranded (ss) RNA; viral ssRNA is an intrinsic component of WIV, but not of other influenza vaccines [6], [15], [30], [31]. Our current study therefore identifies viral ssRNA as the key component of WIV, not only for the induction of superior humoral immune responses dominated by the Th1-related antibody subtype IgG2a/c as described earlier, but also for the induction of cellular immune responses with cross-protective potential [15].

The observation that DCs cannot cross-present WIV-derived antigens (e.g. NP-derived epitopes) in the absence of TLR7 signaling is a key mechanistic finding. Successful cross-presentation of exogenous antigens necessitates that DCs acquire a mature and activated phenotype, characterized by upregulated expression of MHC class I and costimulatory surface markers, and by the production of specific cytokines. Immature DCs, as well as DCs that have already matured before target antigen encounter, fail to induce CTL activation [25], [32]. One way by which DCs can acquire a mature phenotype is through direct recognition of pathogen-associated molecular patterns (PAMPs) by innate pattern recognition receptors (PRRs) [23], [33], [34]. As mentioned above, viral ssRNA is an integral component of WIV and, as a natural ligand for TLR7, a potent activator of DC maturation [30]. In the present study, we show that stimulation of DCs with WIV induced maturation and activation in a strictly TLR7-dependent fashion. This explains the complete failure of TLR7−/− DCs to cross-present WIV-derived antigens.

Notably, WIV induced maturation and activation of isolated pDCs; this was not apparent for cDCs in isolation. However, in mixed cultures, cDCs also acquired a more mature phenotype, likely in response to cytokines produced by activated pDCs (Fig. 7). pDCs were also important for CTL induction upon WIV immunization *in vivo,* as indicated by reduced numbers of CTLs and impaired protection in mice immunized after depletion from pDCs (Figs. 9, 10). It has to be mentioned that virus titers in pDC-depleted mice were lower than in mock-immunized control mice and these mice were partially protected from severe weight loss. This could be either due to the residual pDCs (since depletion was about 70 but not 100%). Alternatively, it might point to the fact that other DC populations also participate or can take over in absence of sufficient pDCs. Whether pDCs or cDCs are the primary cell population cross-presenting WIV-derived antigens *in vivo* remains unclear. Generally, CD8α+ lymphoid-organ-resident cDCs are considered to be the major cell population in mice capable of cross-presentation, followed by migratory CD103+ DCs and inflammatory DCs [35]–[37]. pDCs are generally considered to be poor APCs but are probably capable of cross-presenting exogenous antigen and priming naive CD8+ T cells when activated with the help of TLR ligands [37]–[40]. Kratky *et al*. demonstrated that direct activation of the APC, e.g. via TLRs, is required for successful induction of CTL responses [41]. This would point to pDCs as the most likely cross-presenting cell population since these cells, in contrast to cDCs, could be activated directly by WIV. However, the Kratky study was performed *in vitro* in the absence of T helper cells capable of activating DCs through CD40 ligation, and is therefore of limited predictive value for the situation *in vivo*. Taken together the available evidence suggests that the ssRNA component of WIV directly activates pDCs through TLR7. Thereby the pDCs are either themselves licensed for cross-presentation of WIV-derived antigens or they are activated to produce factors that license cDCs for doing so.

After activation by WIV, pDCs produce substantial amounts of IFNαand IL12, both previously described to be essential for cross-priming [42]. Type I IFNs, like IFNαenhance DC recruitment to draining lymph nodes, prevent early antigen degradation and promote routing of antigen to proper processing compartments [42]–[44]. IL12, though not affecting the process of cross-presentation in DCs, is an important factor in functional maturation of CTLs activated by cross-presenting DCs [42].

Thus, in addition to a possible direct role in cross-presentation, pDCs likely contribute to influenza-specific CTL induction in response to WIV via secretion of type I IFN and IL12, both known to be intricately involved in CTL activation. Also in the context of induction of antibody responses, and particular the Th1 dominance of these responses, pDC-derived cytokines, e.g. type I IFN, were shown to be crucially involved [15], [28].

Our finding that TLR7 activation is critical for the induction of influenza-specific CTL responses by WIV illustrates how a TLR ligand, in this case ssRNA as an intrinsic component of the vaccine itself, can act as an adjuvant. There are other examples of how the CTL-inducing capacity of protein-based vaccines can be dramatically improved by the introduction of TLR ligands. Jelenik *et al*. showed that dsDNA (TLR3 ligand) facilitates CTL induction by a NP-based experimental influenza vaccine [45]. Furthermore, Wagner *et al*. demonstrated that the capacity of split vaccine to induce protective responses mediated by CTLs could be improved by supplementation with CpG (TLR9 ligand) [46]. Interestingly, in contrast to these vaccines, influenza WIV already contains ssRNA as an integral “built-in” adjuvant.

In conclusion, the results of the current study show that in mice TLR7 signaling plays a crucial role in the induction of cross-protective CTLs by vaccination with WIV. Activation of the innate receptor underlies the capacity of this classic influenza vaccine to provide protection against infection with heterosubtypic influenza viruses. Viral ssRNA, an integral component of WIV, is a natural ligand of TLR7 in mice and of TLR8 in humans and represents a potent intrinsic adjuvant in this vaccine formulation. Plasmacytoid DCs are the primary target cells for WIV and probably contribute in multiple ways to the induction of cross-protective cellular immunity. These insights provide a lead for the further improvement of WIV-based vaccines as well as for the development of other CTL-inducing vaccines.

## Supporting Information

Figure S1 **To verify successful depletion of pDCs bone marrow was isolated from the femur of mice one day after the administration of the last dose of buffer, control antibody or the pDC-depleting antibody 120G8.** Cells were stained with antibodies specific for CD11b, CD11c and B220 or PDCA-1. Cells were analyzed by flow cytometry. Gates were set on viable cells based on the forward/side scatter profile and exclusion of 7AAD+ cells. Next gate was set on CD11b− cells and pDCs were identified as CD11c+PDCA1+ or CD11c+B220+.
(TIFF)
