# Supplementary figures and images for: Critical Role of TLR7 Signaling in the Priming of Cross-Protective Cytotoxic T Lymphocyte Responses by a Whole Inactivated Influenza Virus Vaccine
Source: PLoS One. 2013 May 2;8(5):e63163. doi: 10.1371/journal.pone.0063163 (PMC3642048; doi:10.1371/journal.pone.0063163)

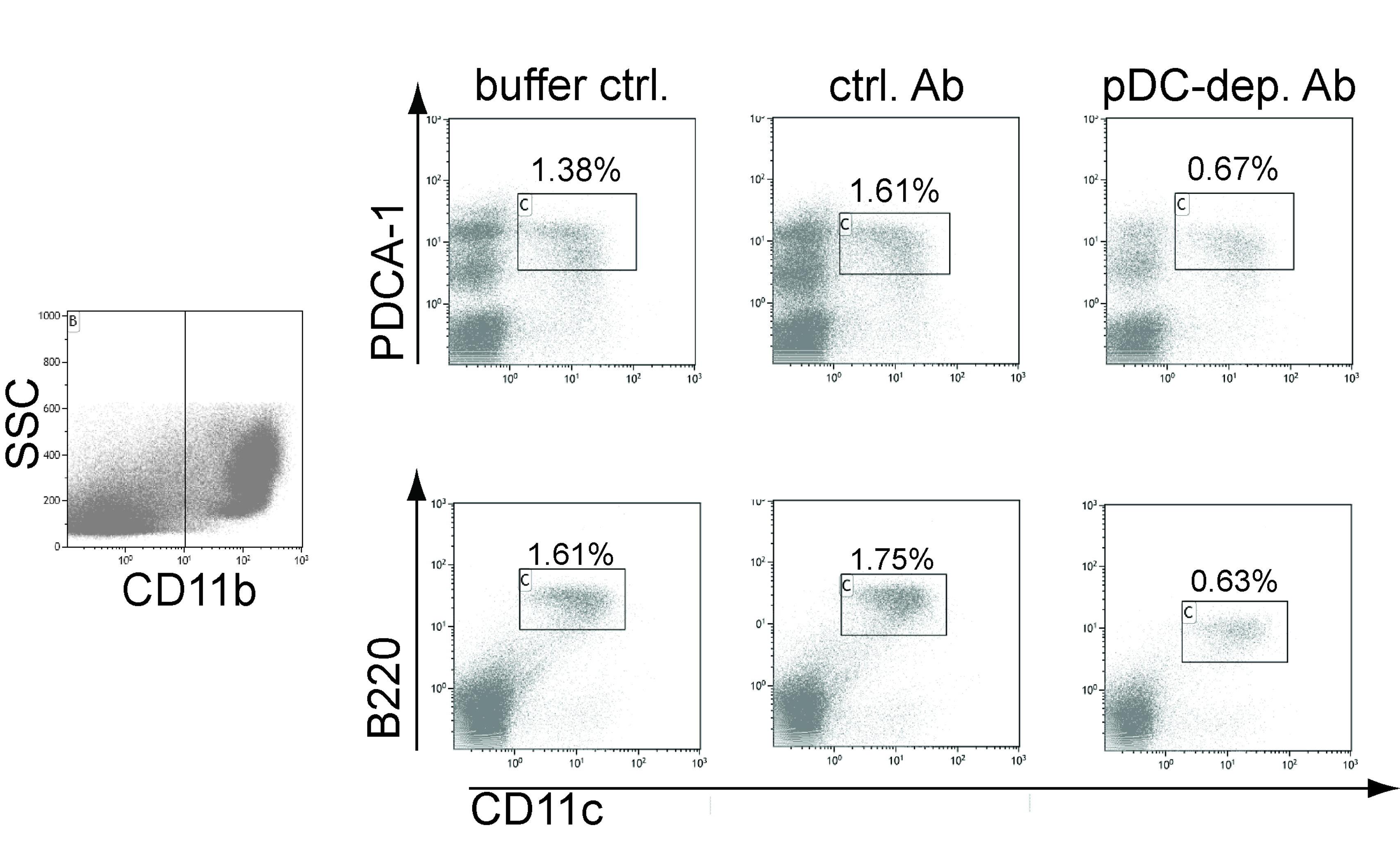

Supplement: Figure S1 — To verify successful depletion of pDCs bone marrow was isolated from the femur of mice one day after the administration of the last dose of buffer, control antibody or the pDC-depleting antibody 120G8. Cells were stained with antibodies specific for CD11b, CD11c and B220 or PDCA-1. Cells were analyzed by flow cytometry. Gates were set on viable cells based on the forward/side scatter profile and exclusion of 7AAD+ cells. Next gate was set on CD11b− cells and pDCs were identified as CD11c+PDCA1+ or CD11c+B220+. (TIFF) [file pone.0063163.s001.tiff]
